# Potential tactics with certain gut microbiota for the treatment of unresectable hepatocellular carcinoma

**DOI:** 10.37349/etat.2023.00152

**Published:** 2023-08-24

**Authors:** Sayuri Yoshikawa, Kurumi Taniguchi, Haruka Sawamura, Yuka Ikeda, Tomoko Asai, Ai Tsuji, Satoru Matsuda

**Affiliations:** Vall d’ Hebron University Hospital, Spain; Department of Food Science and Nutrition, Nara Women’s University, Kita-Uoya Nishimachi, Nara 630-8506, Japan

**Keywords:** Hepatocellular carcinoma, non-alcoholic fatty liver disease, metabolic-associated fatty liver disease, gut microbiota, probiotics, immune checkpoint inhibitors, T helper 17 cells, regulatory T cells

## Abstract

Hepatocellular carcinoma (HCC) constitutes an extremely malignant form of primary liver cancer. Intricate connections linking to the immune system might be associated with the pathogenesis of HCC. Meanwhile, immunotherapy with immune checkpoint inhibitors has been established to be a favorable therapeutic possibility for advanced HCC. Although curative opportunities for advanced HCC are restricted, the immune checkpoint immunotherapy has developed as the main choice for treating HCC. However, patients with metabolic-associated fatty liver disease (MAFLD)-linked HCC might be less likely to benefit from the immunotherapy alone. The limitation of the effect of the immunotherapy might be owing to the impaired T cell activation in MAFLD patients, which could be well explained by a dysfunctional gut-liver axis. Gut microbiota and their metabolites including several bile acids could contribute to modulating the responses of the immune checkpoint immunotherapy. Roles of gut microbiota in the development of cancers have expected great interest in the latest studies. Here, an interplay between the gut and liver has been presented, which might suggest to affect the efficacy of immune checkpoint immunotherapy against HCC.

## Introduction

Hepatocellular carcinoma (HCC) is the regular type of primary liver cancer approximately constituting 80% of cases [1]. HCC is an aggressive malignancy, which has been a healthcare burden worldwide [1]. Therapies for HCC are determined by the clinical stages of the disease. In case of early stage HCC, localized therapies such as ablation, radiation, and resection including hepatectomy are the typical treatment [2]. Among them, surgical management is the most imperative method for HCC patients to achieve long-term survival. In fact, the guidelines for the treatment of primary liver cancer had proposed surgical resection as the first choice for the treatment of HCC in stages with a spare of liver functional ability. However, these patients only represent about 20% of the total. In addition, the 5-year survival rate may not be satisfactory with more than 70% of patients unfortunately relapsing within 5 years [3]. Furthermore, HCC is frequently diagnosed in advanced and/or unresectable stages [4]. Therefore, HCC has high mortality and a high risk of recurrence even after drastic treatment. The majority of the patients with HCC diagnosed at advanced stages may miss the ideal time for remedial management. Then, there are few therapeutic treatments with limited possibilities and limited survival benefits, which is making the cure rate quite low [5]. At present, chemotherapy treatments for HCC are largely separated into targeted therapeutic medications and immunotherapeutic agents. Tactlessly, systematic palliative treatment may be also the option for most patients with advanced-stages of HCC [6].

**Figure 1:**
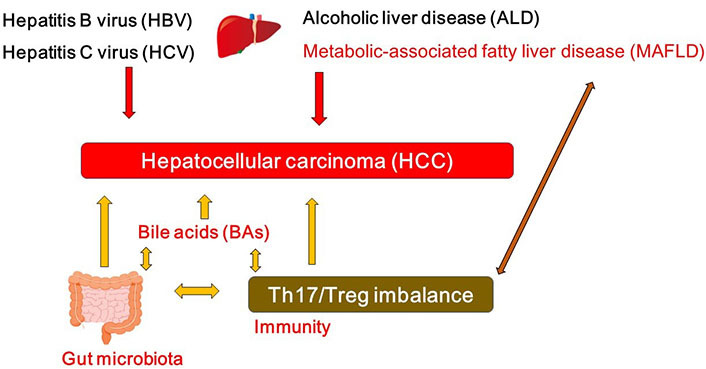
A hypothetical schematic representation and overview of the pathogenesis of HCC. HBV, HCV, ALD, and/or MAFLD as well as bile acids (BAs), T helper 17 (Th17)/regulatory T (Treg) imbalance, and/or gut microbiota could contribute to the pathogenesis of HCC. Arrowhead indicates stimulation whereas double-arrowheads suggests bidirectional stimulation. Note that several important activities such as cytokine-induction or anti-inflammatory reaction have been omitted for clarity

While viral hepatitis with hepatitis virus such as hepatitis B virus (HBV) and/or hepatitis C virus (HCV) may characterize one of the most significant reason for HCC, alcoholic liver disease (ALD) and metabolic-associated fatty liver disease (MAFLD) are also the leading cause for the development of HCC (Figure 1). In particular, MAFLD may be a leading premonition of HCC [7]. The combination of a high fat diet and bacterial endotoxin could contribute to the activation of both innate and adaptive immune responses, which may be headmaster to the pathogenesis of MAFLD and HCC [8]. MAFLD-associated inflammatory, metabolic and/or endocrine mediators might play key roles in several tumorigenesis. However, the carcinogenesis to HCC may be multi factorial compelled by several other chronic inflammation such as colitis and/or pancreatitis. Major risk factors for HCC may also contain other metabolic disorders such as diabetes, liver fibrosis, aflatoxin-induced liver toxicity and immune-related diseases including autoimmune hepatitis [9, 10]. In addition, an imbalance in the composition of gut microbiota may lead to a disrupted intestinal barrier, which could direct to the translocation of bacteria and/or their products into the portal circulation, then increase hepatic exposure to harmful substances that might afterward result in chronic inflammation and/or the development of MAFLD or HCC [11, 12]. In other words, the balance of the gut microbiota might be indispensable for a physiological and appropriate functioning of the intestinal barrier to avoid the development of MAFLD or HCC.

## HCC and gut microbiota

Emerging evidence implicates a key role of gut microbiota in liver inflammation and the progress of HCC [11]. The gut-liver axis is the key mechanism by which the gut microbiome promotes various liver disease and/or HCC [12]. For example, *Anaerotruncus* enriched in the case of antibiotics usage are butyrate-producing bacteria with a positive correlation to the intake of saturated fatty acids and cholesterol, which may be involved in MAFLD-associated HCC [13]. Dysfunction of gut microbiota might provoke a neutrophil accumulation into the gut epithelium that alters the composition of inflammatory cytokine and/or chemokine, which might stimulate the Th17 cells [14]. The intra-tumoral regions of HCC are generally in an immunosuppressive state [15], which may include altered amount of effector to Treg cells and a profusion of immunosuppressive molecules developing a linkage to assist immune escaping [16]. Likewise, increased levels of Th17 cells have been observed in tumor tissue [17] and/or even in peripheral blood [18] of the patients with HCC, however, which might be interrelated with unfavorable disease consequences [17, 19]. Similar results have been also detected in animal models, whereby restrictive expansion of Th17 cells in tumor may reduce the growth of the transplanted liver tumor in animal models [20]. The gut might be an expected place for Th17 generation. It has been shown that gut microbiota could affect T cell differentiation of Th17 cells via regulating dendritic cells [21]. As a consequence, there appears to be a complex association among gut microbiota, the development of Th17 cells and the progression of HCC (Figure 1).

The source of tumor-associated Th17 cells may be also connected to the gut [22]. In addition, Th17 cells seem to be intensely linked with HCC progression, probably via assisting their angiogenesis [20, 23]. Interestingly, probiotic alteration of the gut microbiota could help prevent the occurrence of HCC [24]. Hence, the shift in the function of gut microbiota might accidentally support tumor formation and growth. In fact, bacterial metabolites in gut are known that play a role in carcinogenesis [25, 26]. Furthermore, modulation of gut microbiota may represent an effective procedure for strengthening anti-cancer immunity, which is suggested by various information that exhibit a close connection between gut microbiota and the pathogenic mechanisms of HCC [27]. The use of probiotics and/or the fecal microbiota transplantation (FMT) method could develop new paradigms with the potential effectiveness of several treatments available for HCC. In particular, Th17 cells seem to be an innovative therapeutic target against tumor-promoting inflammation. Strategies using probiotics and/or FMT to polarize the response away from Th17 cells might be useful to slow down the tumor growth of HCC.

## HCC and BAs

Microbial products could closely affect the function of liver, whose derivatives including BAs could lead to carcinogenesis in liver [28]. Connection between BAs and HCC is progressively being established by several experiments. For example, the accumulation of toxic BAs might be related to the progression of HCC (Figure 1) [29]. In addition, experiments in which mice fed with a high-fat diet exhibit that long-term high-fat diet could induce liver tumors in mice along with the observation of considerably increased BAs in plasma and/or in liver [30]. BAs may be vigorously changed all the way to the impairment of the animal in the course of HCC progression. BA molecules have hydrophobic regions and hydrophilic regions in their configuration, which might provide them with interfacial activity to decrease the surface tension [31, 32]. In addition, BAs can extend the contact surface between lipids and lipase to speed up the digestion of various lipids [31]. BAs can also prevent the precipitation of cholesterol to inhibit the formation of cholesterol stones [31, 32]. Since BAs are great surfactants, bacteria in gut have to protect themselves against being degenerated by BAs. Accordingly, commensal bacteria in gut may have a complicated system to survive with BAs [32]. In addition, connections between BAs and gut microbiota may be bidirectional. For example, gut microbiota could convert some of the primary BAs to modulate the composition of the BAs [33]. In reverse, BAs could also affect the composition of the gut microbiota (Figure 1) [34]. Hence, BAs might work as potent controllers of gut microbiota [35]. Dysbiosis of gut microbiota associated with several cancers [36] could change the composition of BAs, which might eventually contribute to the carcinogenesis (Figure 1) [37, 38]. Oxidation and/or epimerization through the reactions of BAs might be interconnected to Firmicutes and/or *Bacteroides* species in the gut [39, 40].

Cell membrane perturbation by BAs might activate phospholipase A2 with the release of arachidonic acid from the plasma membrane, eventually leading to the production of increased levels of reactive oxygen species (ROS) in hepatocytes [41]. Therefore, BAs could damage the cell membrane, eventually leading to the increased levels of inflammation [41]. Apoptotic cells as a result of BAs might also initiate the inflammation. ROS may induce DNA damage in hepatocytes and contribute to the occurrence of HCC [42]. BAs could induce genetic instability scratched by the DNA damage via oxidative stress [43]. Therefore, the role of BAs has been involved in a wide range of cancers including HCC. For example, hydrophobic BAs could contribute to the growth of HCC [44]. In addition, elevated BAs in serum for a long-term has been identified in HCC patients by metabolomics [45]. Furthermore, conjugated BAs such as taurocholic acid and/or taurodeoxycholic acid could promote the progression of esophageal adenocarcinoma [46]. Acidic bile salts could stimulate epithelial to mesenchymal transition in Barrett’s cells [47]. High concentrations of BAs might be associated with an elevated risk of intestinal metaplasia [48], which could increase the risk of gastric cancer [49]. Additionally, BAs might be also involved in the initiation and/or the progression of pancreatic cancer. In fact, several BAs have shown an extreme increase in the patients with pancreatic cancer at multiple stages [50]. In pancreatic cancer cells, BAs could reduce the susceptibility of cancer cells to lead to apoptosis [51]. It has been shown that the damaging effect of BAs on colon epithelial cells might induce a compensatory renewal mechanism of the epithelium by inducing the epithelial cells to convert cancer stem cells [52]. It has been reported that both lithocholic acid and deoxycholic acid have tumor promoter activity on the cells at crypt of colon in the early phase of carcinogenesis [53]. In summary, BAs could contribute to the carcinogenesis of various cancers including HCC.

## HCC treated with immune checkpoint inhibitors

Immune checkpoint inhibitors have been permitted for clinical usage for HCC treatment, which have revealed some efficacy in many clinical trials afterwards [54]. Up until now, cytotoxic T-lymphocyte associated protein 4 (CTLA-4), programmed cell death 1 (PD-1), programmed cell death ligand 1 (PD-L1) and/or micro-environmental immune cells seem to be associated with the effectiveness. Each treatment appears definite efficacy and/or small toxicity profiles, being related to the tumor microenvironment of HCC [55]. By discharging the immune checkpoints which standstill the function of T-cells, each treatment could result in the re-activation of an anti-cancer immune system efficiently to attack the cancer cells [56]. As for advanced stages of HCC patients, a nearly complete response such as down-staging has been achieved by those treatments with immune checkpoint inhibition, while actual few has detected non-lethal and/or reversible adverse events [57, 58]. In addition, immune checkpoint inhibitors seem to increase overall survival among patients with advanced stages of HCC. More strategies should embrace a combination of immune checkpoint inhibitors with additional therapies to achieve the more vigorous responses [59].

The pathophysiological association among the PD-1/PD-L1 pathway, Th17 cells and Treg cells has been shown, suggesting an indispensable role of PD-1/PD-L1 in the regulation of Th17/Treg cells [60]. Similarly, CTLA-4 inhibitor therapy could also enhance Th17 cells [61]. In addition, blockade of CTLA-4 could also inhibit Treg cells [62]. Surprisingly, IL-17 and its primary source Th17 could upregulate PD-L1 expression and may hamper the efficacy of the immunotherapy [63]. Th17 cells are resistant to steroid therapy [64]. Similarly, the Th17 cells seem to be always associated with poor responses in cancer immunotherapy. In the mechanisms of immune checkpoint, interaction of PD-1 with PD-L1 might cause a reduction of phosphoinositide 3-kinase (PI3K) expression in T lymphocytes leading to the induction of Treg cells [65]. Th17 cells can also trans-differentiate into suppressive Treg cells [66], providing a source of tumor-associated Treg cells. On the contrary, elevated expression of PI3K/protein kinase B (AKT)/mammalian target of rapamycin (mTOR) molecules may induce a conversion of Treg cells into Th17 cells in the situation of PD-1/PD-L1 dysfunction [65]. Hence, when the PD-1/PD-L1 pathway is congested by immune checkpoint inhibitors, the mTOR pathway might be activated. Accordingly, Th17 cells are abundant and Treg cells are less present. Excessive inflammation from Th17 cells may play significant consequences in several inflammation-associated carcinogenesis [17, 67]. Flexibility of Th17 cells according to the situation might provide a valuable strategy to enhance cancer immunotherapies.

This immune checkpoint inhibitory immunotherapy is deliberated a step frontward to the management of a diversity of malignancies, particularly in advanced stages of cancers. The immunotherapy could be pretty specific to the cancer cells without showing any negative effects on healthy cells and/or organs. The molecular mechanism is targeting at specific antigens presented in malignant cancer cells [68]. Subsequently, the research strategies to overwhelm the resistance of cancer cells seem to be heading toward an intensified strategy comprising better combination with different agents such as PD-1 plus CTLA-4 blockades. The gut-liver axis might be an anatomical and physiological connection between the liver and gut, which may help not only to maintain normal liver functions but also to protect the liver from the development of carcinogenesis [24]. Therefore, exposure of the liver to chronic lesions to bacterial metabolites may result in liver damage and eventually the development of HCC [24].

## Therapeutic approach for patients with unresectable stages of HCC

**Figure 2:**
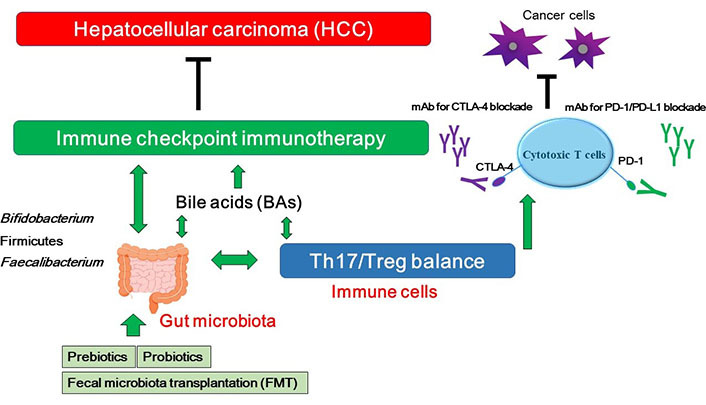
A hypothetical schematic representation and overview of the inhibition against HCC. BAs, Th17/Treg balance, and/or certain gut microbiota could contribute to the potentiation of the immune checkpoint immunotherapy with immune checkpoint inhibitors. Some kinds of probiotics, prebiotics, and FMT could contribute to the alteration of gut microbial community for playing valuable roles to the immune checkpoint therapy. Examples of certain beneficial microbial species with several effects on anti-cancer immune responses have been shown at the left side. Arrowhead indicates stimulation whereas double-arrowheads suggests bidirectional stimulation. Hammerhead shows inhibition. Note that several important activities such as cytokine-induction and/or anti-inflammatory reaction have been omitted for clarity. mAB: monoclonal antibody

As mentioned above, dysbiosis may influence the effectiveness of anticancer therapies including immune checkpoint inhibitors in some types of patients with cancers. Consistently, the intestinal microbiota could increase the effectiveness and/or the sensitivity of the treatment with anti PD-1 against HCC [69, 70], which suggests that the modulation of gut microbiota can manage to increase the activity of HCC treatments. Microbiota of patients who have responded to PD-1 immunotherapy exhibits increased certain gut bacterial species [70, 71]. In addition, germ-free mice transplanted with fecal samples from patients responding to anti-PD-1 and/or anti-PD-L1 immunotherapy have showed a reduction in the tumor growth and/or enhanced responses to anti-PD-1 and/or anti-PD-L1 treatment [71]. Reliably, the gut microbiota can affect the efficacy of immune checkpoint immunotherapy [72]. Specific bacteria possibly for enhancing the immune-stimulatory anti-cancer effects of PD-1 and/or CTLA-4 blockades have been shown in Table 1. In addition, it has been reported that modulation of gut microbiota may form a strong procedure of manipulation for the anti-tumor immunity [73]. Patients with immunoresistance to anti-PD-1 treatment of HCC may exhibit an enlarged amount of *Ruminococcus* spp. and *Akkermansia muciniphila* in their fecal samples [69]. In addition, intake of *Bifidobacterium* could improve the response to PD-L1 immune checkpoint immunotherapy against HCC [74]. Similarly, it has been reported that intake of Firmicutes and *Faecalibacterium* could augment the response to anti-CTLA-4 antibodies [74]. Interestingly, FMT in combination with oral supplementation of *Akkermansia muciniphila* could change the immune-resistance to the PD-1 immunotherapy [75]. Probiotics, prebiotics and FMT could shift the gut microbial community toward certain beneficial bacteria, which subsequently decreases the Th17 polarization and/or promotes the differentiation of anti-inflammatory Treg cells leading to slow down the growth of HCC tumor (Figure 2) [76]. On the other hand, microbial dysbiosis could direct to the disruption of the gut barrier, which may influence the production and construction of BAs, resulting in a carcinogenesis of a broad spectrum of malignant tumors including HCC [41]. In addition, the changed components of BAs might be connected with immune-resistance [77]. It has been shown that BAs might be enriched in cancer patients, which is associated with poor prognosis [78]. BAs could alter the gut microbial population [79], indicating that BAs might also affect the efficacy of immune checkpoint immunotherapy by gut bacterial alteration (Figure 2).

**Table 1:** Microbial species which may enhance the effect of immune-checkpoint cancer therapies

**Immunotherapies**	**Microbial species**	**References**
Anti PD-1/PD-L1 blockade	*Bacteroides cellulosilyticus* *Bifidobacterium dentium* *Coprococcus comes* *Lactobacillus oris* *Lactobacillus mucosae* *Streptococcus thermophilus*	[69, 74]
Anti CTLA-4 blockade	*Bacteroides thetaiotaomicron* *Bacteroides fragilis* *Burkholderia cepacia* *Faecalibacterium genus*	[74, 101]

Provided several significant results acquired from immunotherapy in patients with advanced HCC [69], it might be imperative to search the data relating to the gut microbiota whether there are the beneficial effects of the immunotherapy against HCC, or not. In addition, the possible modulation of the gut microbiota could help in overcoming any resistance to immunotherapy in patients with MAFLD-linked HCC [80]. Both fecal microbiota and BAs are related to consequences of immunotherapy for HCC [81]. These findings emphasize the possible role of gut microbiota and their metabolites to predict the consequences of immune checkpoint immunotherapy-treated HCC. Interestingly, a curcumin analog controls the metabolism of both Treg cells and tumor cells and could serve as a booster for immune checkpoint inhibitor therapies [82]. In the attempt to intensify the efficacy of an immunotherapy for HCC, double or triple checkpoints blockade such as anti PD-1 and anti CTLA-4 or anti PD-L1 and multi-target tyrosine kinase inhibitors have been suggested [83].

## Future perspectives

The management of HCC has been radically changing. Usage of tyrosine kinase inhibitors and the introduction of the immunotherapy might improve the survival and the progression free period of the HCC even in advanced stages [84]. However, the efficacy of those strategies may be empowered by an adapted patient selection. Since patients with HCC are disposed to have more or less changes in gut microbiota, it may be striking to suppose that some modulation of gut microbiota could affect the efficacy of anticancer treatments in certain types of patients. And so, distinct bacterial species could control different immune responses. Gut microbiota-derived metabolites such as BAs and/or short-chain fatty acids (SCFAs) may be also involved in the regulation of inflammation and/or carcinogenesis [85]. In addition, these changes could be associated with an inflammatory and/or immune power shift. The life-style diet may be responsible for the dysbiosis, which is an important risk factor for carcinogenesis and/or influences the therapeutic outcomes, as for various anticancer treatments including the immune checkpoint immunotherapies [86]. In fact, several findings demonstrate that gut microbiota could affect the response to the immunotherapy as mentioned earlier. However, some bacterial products such as lipopolysaccharide (LPS) may stimulate the toll-like receptors (TLRs), in particular TLR-4, which in turn can activate the nuclear factor kappa B (NF-κB) pathway [87, 88]. The TLR-4/myeloid differentiation factor 88 (MyD88) pathway has been known as oncogenic signaling in human HCC and may be correlated with patients’ poor survival [89]. The immunomodulatory signaling with TLR-4 could induce the stimulation of mitogen-activated protein kinase (MAPK) following a mitogenic signal [89], which may be associated with an inhibition of programmed cell death. Therefore, prolonged stimulation of several TLRs with various bacterial substances in hepatocytes could promote the development of chronic liver diseases and/or HCC [90]. Consequently, the orientation of research towards the use of prebiotics, probiotics and/or FMT should guide to a new personalized treatment-paradigm with understanding the precise roles of gut microbiota for the more effective treatments available to HCC [91]. Prebiotics and/or probiotics could have a therapeutic effect against the chronic inflammation related cancers [91]. It remains to be clarified whether the gut microbiota in some types of malignant tumors could also be practical to the patients with HCC. Commensal bacteria are fundamental in coordinating antitumor responses in various tumor microenvironment [92–94]. In addition, BAs could also regulate adaptive immune responses by directly modifying the balance of Th17 cells and Treg cells, suggesting much wide-ranging roles of BAs in regulating inflammation and/or cancer [95–97]. Insertion of immune checkpoint inhibitors with the other combination therapy might achieve the benefit of therapeutic efficacy within this research approach, as this would allow patients to be directed to the most appropriate and safe treatment. For example, several objective therapeutic strategies for patients with unresectable advanced HCC have been proposed [98–100], which appear to be quite promising for the combination therapy with this immune-potentiation therapy. Despite convincing evidences regarding the roles of the gut-liver axis in the pathogenesis of HCC, however, putting this fact into a clinical practice is still a work in slow progress.

## Conclusions

The tumor microenvironment of HCC might be intricate and dynamic. In some cases, the interplay between liver and gut could affect the efficacy of several anti-cancer treatments including immune checkpoint immunotherapy via the alteration of Th17 cells. Come to think of it, complicated interactions among HCC cells, suppressive immune cells, and the gut microbiome might produce a permissive microenvironment that facilitates immune evasion to approve HCC growth.

## Abbreviations

ALD: alcoholic liver disease
BAs: bile acids
CTLA-4: cytotoxic T-lymphocyte associated protein 4
FMT: fecal microbiota transplantation
HBV: hepatitis B virus
HCC: hepatocellular carcinoma
HCV: hepatitis C virus
MAFLD: metabolic-associated fatty liver disease
PD-1: programmed cell death 1
PD-L1: programmed cell death ligand 1
Th17: T helper 17
TLRs: toll-like receptors
Treg: regulatory T

## Declarations

### Copyright

© The Author(s) 2023.
